# Parental reports of the oral health-related quality of life of children with cerebral palsy

**DOI:** 10.1186/1472-6831-12-15

**Published:** 2012-06-18

**Authors:** Jenny Abanto, Thiago S Carvalho, Marcelo Bönecker, Adriana OL Ortega, Ana L Ciamponi, Daniela P Raggio

**Affiliations:** 1 Pediatric Dentistry and Orthodontics Department, Dental School, University of São Paulo-USP, São Paulo, Brazil

**Keywords:** Cerebral palsy, Children, Oral health related quality of life

## Abstract

**Background:**

The severity of physical and mental impairments and oral problems, as well as socioeconomic factors, may have an impact on quality of life of children with cerebral palsy (CP). The aim of this research was to assess the impact of impairments and oral health conditions, adjusted by socioeconomic factors, on the Oral Health-Related Quality of Life (OHRQoL) of children with CP using their parents as proxies.

**Methods:**

Sixty children, between 6-14 years of age were selected. Their parents answered a children’s OHRQoL instrument (5 domains) which combines the Parental-Caregivers Perception Questionnaire (P-CPQ) and Family Impact Scale (FIS). The severity of dental caries, type of CP, communication ability, gross motor function, seizures and socioeconomic conditions were assessed.

**Results:**

Considering the total score of the OHRQoL instrument, only the reduction of communication ability and dental caries severity had a negative impact on the OHRQoL (p < 0.05). Considering each domain of the instrument, the severity of the type of CP and its reduction of communication ability showed a negative impact on oral symptoms and functional limitations domains (p < 0.05). Seizures have a negative impact on oral symptoms domain (p = 0.006). The multivariate fitted model showed that the severity of dental caries, communication ability and low family income were negatively associated with the impact on OHRQoL (p = 0.001).

**Conclusions:**

The severity of dental caries, communication ability, and family income are conditions strongly associated with a negative impact on OHRQoL of children with CP.

## Background

Cerebral palsy (CP) is a neurological disorder occurring in approximately 2 to 2.5 per 1000 live births [1]. CP describes a group of disorders of movement and posture. Some motor disorders may be accompanied by disturbances of sensation, cognition, communication, perception and seizures [2].

Studies have shown that the more severe the neurological damage in children with CP, the higher the risk of oral diseases [3,4]. This occurs not only because of their dietary consistency and the greater difficulty that these individuals have to move and perform, or receive, an effective oral hygiene, but also to the limited oral care that this population is exposed.

Good quality of life (QoL) is a key outcome for the individual child and is what society wants for all children [5]. The concept of OHRQoL relates to the impact which oral health or disease has on the individual’s daily functioning, well-being or life quality [6]. However, to our knowledge, there are no studies focusing on the impact of impairments and oral health conditions on the Oral Health-Related Quality of Life (OHRQoL) of children with cerebral palsy. Besides that, some studies have also reported that socioeconomic factors have direct influence on oral health and on the responses about impact of several diseases on the quality of life [6-8]. The magnitude of this effect in children, however, remains unclear.

In view of the scarce studies assessing OHRQoL in patients with cerebral palsy, as well as the influence of socioeconomic factors in perception of quality of life; the purpose of this study was to assess the impact of dental caries and health conditions, such as type of CP, communication, motor ability and presence of seizures adjusted by socioeconomic factors on the OHRQoL of children with CP.

## Methods

This study was independently reviewed and approved by the ethical board of the Dental School–University of São Paulo (Brazil).

### Sampling and procedure

For this cross-sectional study, a convenience sample was selected. It comprised children with CP who attended the Center of Attendance for Special Needs Patients - CAPE of the Dental School, University of São Paulo. The CAPE performs preventive and restorative treatment for individuals with different types of disabilities.

The sampling was conducted in two stages: initially, all children from 6 to 14 years of age, of both genders in the database system at the center were identified, resulting in a total of 75 children. The patients’ parents were contacted by phone and informed about the study. From a total of 75 children, 5 could not be contacted because they have moved, 2 children have died, 3 were hospitalized and from 5 of them, the parents did not want to participate in the study. Therefore, the final sample size comprised of 60 children with CP and their respective parents. Parents who were fluent in Brazilian Portuguese and who were willing to participate in the study (all of them) signed an informed consent.

### Data collection

On the day of the dental visit, one of the parents (always the same) was invited to answer two questionnaires: one on the children’s OHRQoL and another on socioeconomic conditions. These questionnaires were collected in face-to-face interviews by one interviewer blind to the oral examinations and impairments characteristics of the child. The interviews were completed before the oral clinical examination of the patient and all parents agree to answers the questions. Concerning the OHRQoL questionnaire, the interviewer was trained in the reading and intonation of each question and option of responses.

### Impairment characteristics of the child

Data were collected by one examiner with experience in children with cerebral palsy. The type of CP was evaluated according to the topographic distribution [9]: hemiplegia, diplegia and quadriplegia. Communication ability was evaluated according to the study of Dickinson et al. [5]: normal; mild disability (some difficulty identified, but uses speech); moderate disability (communication mainly uses non-speech forms) and severe disability (no formal communication is identified). The Gross Motor Functioning Classification System (GMFCS) was evaluated according to Palisano et al. [10]. The parents were also asked if the children have seizures.

### OHRQoL instrument

The OHRQoL instrument used in this study was a 47-item questionnaire which combines two questions on global ratings, the Brazilian version of the Parental-Caregivers Perceptions Questionnaire (P-CPQ) [11] and the Family Impact Scale (FIS) [12] components of the Child Oral Health Quality of Life Questionnaire (COHQOL^©^) [13]. The P-CPQ and FIS were validated in the original language [14,15] and recently transculturally adapted and completely validated in the Brazilian Portuguese language. The interviews for this study were done in February 2009 at the University of São Paulo. This instrument evaluates the perception of parents on OHRQoL of children aged 6 to 14 years. The proxy-report was chosen due to the difficulties to assess self-report in most of these children. The questions were asked in reference to the frequency of events in the previous three months. The items were scored using a five-point Likert scale (response options: never = 0, once or twice = 1, sometimes = 2, often = 3, every day or almost every day = 4). A “Don’t know” response option prevents the loss of valuable information, which would occur if complete data from participants with nonresponse to some items were deleted [14]. Then, the number of “Don’t know” responses was counted individually. Due to the fact that this could affect the data analyses, the “Don’t know” responses were considered as missing in Table 1. Therefore, the mean response in each domain was calculated by adding the total score for the domain and dividing by the number of questions with responses. Some other ways to handle “don’t know” responses are described in Marshman et al. (2007) [16].

**Table 1:** **Mean difference between impairments and dental caries for each domain and for total score - OHRQoL instrument**

**Clinical condition and impairments**	**n(%)**	**OS**	**(± SD)**	**FL**	**(± SD)**	**E-WB**	**(± SD)**	**S-WB**	**(± SD)**	**FIS**	**(± SD)**	**Mean Score**	**(± SD)**
**Type of CP**													
Hemiplegia	24 (40.0)	4.3A	3.0	4.5A	3.6	0.5	1.2	0.9	1.2	3.1	3.4	16.4	9.1
Diplegia	16 (26.6)	4.3A	3.3	4.6A	3.6	1.3	1.8	0.5	0.6	2.6	2.5	15.4	10.1
Quadriplegia	20 (33.4)	9.7B	3.7	7.8B	4.3	1.0	2.8	0.8	1.4	4.2	7.1	20.3	18.7
p – value		<0.001‡		0.011†		0.211†		0.780†		0.965†		0.461‡	
**Communication ability**													
Normal to mild disability	33 (55.0)	4.4	3.4	4.3	3.7	0.4	1.0	0.9	1.3	2.3	2.8	13.2	9.31
Moderate to severe disability	27 (45.0)	8.3	4.1	7.3	4.1	1.4	2.6	0.6	0.9	4.7	6.4	22.7	15.81
p – value		<0.001*		0.005*		0.040 §		0.700 §		0.061*		0.006*	
**Gross Motor Function Classification**													
GMFCS I to III	18 (30.0)	5.0	3.7	4.5	4.4	0.6	1.3	0.8	1.0	2.6	2.4	15.4	9.7
GMFCS IV and V	42 (70.0)	6.5	4.3	6.1	3.9	1.0	2.2	0.7	1.2	3.6	5.5	18.1	14.5
p – value		0.205*		0.166*		0.531 §		0.414 §		0.993 §		0.486*	
**Seizures**													
Yes	18 (30.0)	8.3	4.0	7.1	3.7	1.1	2.4	0.7	1.0	3.9	5.9	19.5	15.4
No	42 (70.0)	5.1	3.9	5.0	4.1	0.8	1.8	0.8	1.2	3.1	4.2	16.3	12.3
p – value		0.006*		0.062*		0.849 §		0.971 §		0.792 §		0.401*	
**Severity of Dental Caries**													
Caries free dmf-t/DMF-T = 0	27 (45.0)	5.9	4.5	4.7	3.4	0.2	0.8	0.7	1.0	1.9A	2.5	12.3 A	8.3
Low severity dmf-t/DMF-T = 1-2	11 (18.3)	6.3	4.2	6.6	3.9	0.4	1.2	1.5	1.4	2.1A	2.9	19.5 A	8.9
Moderate severity dmf-t/DMF-T = 3-4	12 (20.0)	6.3	3.6	5.6	4.5	1.7	1.8	0.6	1.0	4.2 A	2.2	19.1 A	10.2
High severity dmf-t/DMF-T ≥ 5	10 (16.7)	5.9	4.6	6.9	5.3	2.2	3.7	0.4	1.0	7.5 B	9.3	26.3 B	23.7
p - value		0.987 ‡		0.410 ‡		0.008 †		0.144 †		0.007 ‡		0.025 ‡	

OS = Oral symptoms FL = Functional limitations E-WB = Emotional well-being S-WB = Social well-being FIS = Familiar Impact Scale.

* T-test † Kruskal Wallis ‡ ANOVA § Mann-Whitney test.

Different letters (A) mean statistically different results (p < .05).

The following subscale scores were created by summing the responses to conceptually based, discrete subsets of items: *oral symptoms domain* - 6 items; *functional limitations domain* - 8 items; *emotional well-being domain* – 7 items; and *social well-being domain* – 10 items. In addition, scores from 14 items on the impacts of the child’s oral condition on parents and other family members were summed to generate the *family impact scale* (FIS). Two questions on global ratings were also answered by parents in the instrument. One of them on the child’s oral health “How would you rate the health of your child’s teeth, lips, jaws and mouth?” and other on the impact of the oral/orofacial condition on his or her overall well-being “How much is your child’s overall well-being affected by the condition of his/her teeth, lips, jaws or mouth?”. They had a five-point response format from ”excellent” to ”poor” for the child’s oral health and from ”not at all” to ”very much” for the overall well-being.

The total instrument score, and scores for individual subscales (domains), were generated by summing the numerical response options according to the five-point Likert scale. The range score of the instrument range from a minimum of 0 to a maximum of 188, including global ratings. Higher scores mean poorer OHRQoL or vice-versa. Therefore, global and the domains’ scores were presented in the tables, but were independently analyzed and discussed.

### Socioeconomic questionnaire

The parent was also invited to answer a questionnaire on their socioeconomic conditions. It included data on household crowding (inhabitant per room), number of siblings, mother’s and father’s education and family income (as measured in terms of the Brazilian minimum wage - BMW, a standard for this type of assessment, which corresponds to approximately US$ 220.00 per month).

### Dental caries index

The patients’ clinical oral examination was realized for the prevalence of dental caries in an individual. DMFT and dmft are indexes to numerically express the caries prevalence and are obtained by calculating the number of decayed, missing, filled teeth in the primary dentition (dmft) and decayed, missing, filled teeth in the permanent dentition (DMFT). These indexes were described by the World Health Organization [17]. For children with mixed dentition, the caries index was obtained by the sum of the dmft and DMFT scores. The overall measure of dmft and DMFT of the children examined was evaluated separately and together by the sum of the components d + m + f/D + M + F. The dmft/DMFT was categorized according to the severity of dental caries, based on the previously proposed scores [17]: dmft/DMFT 0 = caries free; dmft/DMFT 1-2 = low severity; dmft/DMFT 3-4 = moderate severity or dmft/DMFT ≥ 5 high severity.

The child’s oral examination for dental caries was carried out by one calibrated examiner. This examiner was a specialist in pediatric dentistry previously trained with pictures of clinical cases for the disorders and diseases by a benchmark examiner (A.L.C). The intra-examiner reliability was established by re-examination of 10 (16.67% of sample) patients and the Kappa value for agreement was 0.95. The clinical examinations were performed, after prophylaxis, in a dental unit using an operating light, a 3-in-1 syringe for drying the teeth, plane mouth mirrors and periodontal probes.

### Data analysis

After a descriptive analysis, the total mean scores of the OHRQoL instrument (outcome) and those for the individual domains were analyzed for differences between impairment characteristics and oral health conditions, and socioeconomic factors. For this initial exploratory analysis, parametric and non-parametric tests were used.

Univariate Poisson Regression analysis correlated the total mean score of the OHRQoL instrument to each impairment and oral health conditions and socioeconomic factors. In this analysis, the outcome was employed as a count outcome, represented by the addition of the scores in the instrument, as performed previously [19,20] and rate ratios (RR) and 95% confidence intervals (95% CI) were calculated. Later, a multivariate model was built with several covariates (communication ability, dental caries, household crowding, father’s education and family income), selected by a forward stepwise procedure with p < 0.20 as the cut-off point. A multivariate Poisson regression analysis correlated the outcome to several impairments and oral health covariates adjusted by socioeconomic factors. The covariates were kept in the final model if p <0.05. Statistical analyses used Stata 8.0, 2003 (Stata Corp, College Station, TX).

## Results

A total of 60 patients participated in the study. The response rate was 80% (60-75). Most of the questionnaires were answered by mothers (97%), the remainder were answered by fathers. The mean age (SD) of the children was 9.13 (2.2) and the mean of dmf-t/DMF-T (SD) index was 2.06 (2.6). The number of cases of the communication ability classification and GMFCS were grouped due to the small number of patients in some impairment levels.

Table 2 shows the means and standard deviation of total score, including global ratings scores, and domains; means and standard deviation without excluding and excluding “Don’t know answers”. The possible range, observed range and number of “Don’t know” answers are also described. Table 1 shows the mean difference between specific health conditions for each domain and for the total score of the OHRQoL instrument. When the mean total score of the instrument was analyzed, it could be observed that a reduction of communication ability and the severity of dental caries had a negative impact on OHRQoL (p < 0.05). Considering each domain, there was a significant difference between the types of CP, as well as the communication ability, to the OHRQoL, regarding oral symptoms and functional limitations (p < 0.05). The severity of the communication ability was also associated with a lower OHRQoL in regards to the emotional well-being (p = 0.040). Seizures have shown a negative impact on the children’s oral symptoms (p = 0.006). The severity of dental caries has also shown a negative impact score on the family (p = 0.007) and emotional well-being (p = 0.008). Table 3 shows the mean difference between socioeconomic factors for the total score of the OHRQoL instrument. Fathers’ education level (p = 0.004) and family income (p < 0.001) were variables statistically associated with the outcome.

**Table 2:** Means, standard deviations, ranges and number of “Don't know” answers of the OHRQoL instrument

	**Mean (SD)**	**Mean (SD) without excluding “Don’t know answers”**	**Mean (SD) excluding “Don’t Know answers”**	**Possible range**	**Observed range**	**"Don't know"**
**Total P-CPQ and FIS including global ratings**	17.28 (13.25)	0.38 (0.29) ^d^	0.45 (0.45) ^d^	0 - 188	0-24	339
**Domains**						
Oral symptoms domain (6)	6.05 (4.17)	1.02 (0.71) ^NS^	1.02 (0.71) ^NS^	0 - 24	0 - 15	0
Functional limitations (8)	5.62 (4.07)	0.75 (0.56) ^NS^	0.81 (0.78) ^NS^	0 - 32	0 - 17	17
Emotional well-being (7)	0.85 (1.97)	0.15 (0.32) ^NS^	0.21 (0.61) ^NS^	0 - 28	0 - 9	98
Social well-being (10)	0.75 (1.11)	0.08 (0.11)^e^	0.24 (0.64)^e^	0 - 40	0 - 4	224
Family impact scale (14)	3.32 (4.76)	0.24 (0.34) ^NS^	0.24 (0.34) ^NS^	0 - 56	0 - 24	0

NS = no significant difference (p > 0.05).

d = Paired t-test p = 0.008.

e = Paired t-test p = 0.035.

**Table 3:** Mean difference between socioeconomic factors for total score of the OHRQoL instrument

**Socioeconomic factors**	**n (%)**	**Mean score**	**(± SD)**	**P- value**
**Child gender**				
Male	30 (50.0)	18.8	16.3	0.390*
Female	30 (50.0)	15.8	9.3	
**Household crowding**				
≤ 1.0 inhabitants per room	38 (63.3)	14.5	9.8	0.120 §
> 1.0 inhabitants per room	22 (36.7)	21.7	16.7	
**Number of siblings**				
None	15 (25.0)	13.5	9.3	0.433 ±
One	28 (46.7)	18.0	9.2	
Two or more	17 (28.3)	19.4	20.2	
**Mother's education**				
< 8 years	32 (53.3)	18.4	15.8	0.500*
≥ 8 years	28 (46.7)	16.0	9.8	
**Father's educations**				
< 8 years	42 (70.0)	20.5	13.8	0.004*
≥ 8 years	18 (30.0)	9.7	8.2	
**Familial income**				
≤ One BMW	21 (35.0)	25.3	17.0	< 0.001±
≤ Two BMW	24 (40.0)	15.5	7.7	
≥ Three BMW	15 (25.0)	8.9	7.3	

* T-test ± ANOVA § Mann-Whitney test.

The univariate analysis shows the impairments characteristics and oral health conditions and socioeconomic factors correlated with the outcome variable (Table 4). The final multivariate fitted model comprised only of three covariates: the severity of dental caries, communication ability and family income (Table 5). Family income greater than one BMW had a positive impact on the OHRQoL in children with CP (p = 0.001). The severity of communication ability of the patients showed a negative impact on the patient’s quality of life (p = 0.001), and the presence of dental caries was also associated with a negative impact (p = 0.001) (Table 5).

**Table 4:** Univariate analysis for association between impairments characteristics, dental caries and socioeconomic factors in relation to total score - OHRQoL instrument

**Covariates**	**Robust RR**	**(95% CI)**	P - value *
**Impairments and Oral Health conditions**			
**Type of CP**			
Hemiplegia			
Diplegia	0.96	0.65, 1.41	
Quadriplegia	1.27	0.80, 1.99	0.521
**Cognitive ability**			
Normal to mild disability			
Moderate to severe disability	1.68	1.18, 2.40	0.004
**Gross Motor Function Classification**			
GMFCS I to III			
GMFCS IV and V	1.17	0.80, 1.70	0.411
**Seizures**			
No			
Yes	1.19	0.78, 1.82	0.412
**Dental caries**			
No			
Yes	1.74	1.23, 2.47	0.002
**Socioeconomic factors**			
**Child gender**			
Male			
Female	0.84	0.58, 1.22	0.365
**Household crowding**			
≤ 1 inhabitants per room			
> 1 inhabitants per room	1.50	1.02, 2.20	0.038
**Number of siblings**			
None			
One	1.33	0.90, 1.97	
Two or more	1.43	0.79, 2.59	0.305
**Mother's education**			
< 8 years			
≥ 8 years	0.87	0.60, 1.26	0.471
**Father's education**			
< 8 years			
≥ 8 years	0.57	0.38, 0.85	0.006
**Familial income**			
≤ 1 BMW			
≤ 2 BMW	0.61	0.43, 0.86	
≥ 3 BMW	0.35	0.22, 0.58	< 0.001

Calculated by Qui-square Test.

Robust RR: Robust Rate Ratio.

**Table 5:** The multivariate fitted model of covariates associated to total score of the OHRQoL instrument

**Covariates**	**Robust RR**	**(95% CI)**	P - value *
**Dental caries**			
No			
Yes	1.74	1.26, 2.40	0.001
**Communication ability**			
Normal to mild disability			
Moderate to severe disability	1.62	1.23, 2.12	0.001
**Family income**			
≤ one BMW (reference)			
≤ two BMW	0.53	0.39, 0.72	< 0.001
≥ three BMW	0.45	0.29, 0.72	0.001

* Test Wald Qui-square: p < 0.001.

Robust RR: Robust Rate Ratio.

Regarding the global ratings assessed by parent’s perceptions: 10%, 10%, 35%, 40% and 2% of the parents assessed their children’s oral health as “excellent”, “very good”, “good”, “fair” and “poor”, respectively. On the question whether the overall well-being of their children is affected by the oral/orofacial conditions, 43%, 15%, 20%, 17% and 5% reported "not at all", "very little", "some", "a lot" and "very much", respectively.

## Discussion

This study evaluates the impact of dental caries, health and socioeconomic conditions on OHRQoL of children with CP. To our knowledge, only few studies have assessed the OHRQoL of cerebral palsied children [21,22]. Furthermore, other studies have assessed the influence of socioeconomic conditions on OHRQoL, as well as the perception of parents about dental caries, but these studies are mainly focused on healthy children [6,17,22].

Studies have shown that the severity of cognitive and communication ability increases the difficulty for a child to express his/her feelings [24,25]. Thus, the discomfort caused by oral diseases would not be verbalized, and parents may not seek treatment. In this study, the severity of CP and communication ability had a greatest negative impact on the oral symptoms and functional limitations domains. The items in these domains are described in the original version of the P-CPQ [14]. One relevant item of the oral symptoms domain is concerning the child’s pain in the teeth or mouth, which showed a higher frequency in comparison to the other items in this domain. This oral pain could be present in children with cerebral palsy who experience muscle spasm due to spasticity [24] or severe cognitive impairment [26]*.* The authors have also reported that children with severe cognitive impairments are believed to experience pain frequently, most often due to chronic medical conditions associated with their physical disabilities or medical procedures aimed at managing those conditions. Children with the fewest abilities experience the most pain [24,26].

The oral symptoms’ and functional limitations domains’ items increased the scores in the total OHRQoL instrument. This is in agreement with the results from Baens-Ferrer et al. [21] who showed that before treatment, the domain of oral symptoms was the most affected in children with disabilities. Also, the caregivers in that study, who usually were members of the family, reported a variety of oral symptoms and daily life problems.

Seizure is an important impairment in children with CP. They experience seizures ranging from 28% to 58% [25,27]. In this study, the presence of seizures was associated with a negative impact on the children’s oral symptoms (Table 1). Breau et al. [24] corroborate this finding, stating that seizure is a significant factor causing general pain, including toothache, in children with CP who experience muscle spasm due to spasticity. Therefore, seizures should also be assessed before formulating a dental treatment plan in CP patients. Dentists should be aware of the possibility that pain in a child with CP, as expressed by parental perception, may in fact be linked to the seizure rather than any dental cause. For that reason, a clinical examination should be carried out by the dentist to verify that the pain is not from a dental cause, and then parents should be referred to the child’s medical doctor for the treatment of seizures.

Dental caries was associated with the total score of the OHRQoL instrument in children with CP. As these patients had already sought dental treatment, it could be suggested that they may have a higher score than those who do not seek dental care. On the other hand, only half the patients (n = 33) had dental caries. However, the severity of dental caries in this study was sufficient to produce a negative impact on the total OHRQoL instrument score. When analyzing individual domains of the questionnaire, it was observed that the severity of dental caries had a negative impact on the emotional well-being domain and on the family domain (Table 1). When compared to schoolchildren in general, high caries index can cause a negative impact mainly in the oral symptoms domain, followed by functional limitations, emotional and social well being, respectively [6,18]. The results found in the present study are probably due to a low dmft/DMFT index mean (2.06) found in the patients, or because they had a small number of caries lesions, or the lesions were not deep enough to produce pain, sensitivity or other functional limitation in patients. However, it has been observed in general, that dental caries produce a negative impact on the total score of the OHRQoL instrument as increasing dental caries experience is associated with a poorer OHRQoL [6,18].Therefore, according to our results it may be suggested that the OHRQoL instrument employed in this study may be used in individuals with CP. Du et al.(2010) [22] also suggested that an instrument that specifically measures OHRQoL would better express the impact of oral diseases.

Oral diseases present in children with special care needs may also have an impact upon the family, as caregivers have reported quality of life concerns which could be attributed to their child’s oral health [20]. A recent study focused on children with cerebral palsy’s caregivers also showed that caregivers have a low quality of life due to the difficult task they have in caring for the oral health and preventing dental caries in these patients [28].

The emotional well-being domain was negatively affected in this study. The severity of cognitive ability increases the difficulty for the children to express their feelings and discomfort, and it often creates a sense of uncertainty and frustration in their parents [24,25]. Further studies have to be done to assess how intense these feelings and perceptions are in children with cerebral palsy.

Surveys have shown that a lower income is related to a poorer oral health [29,30]. So, it is expected that the quality of life is also related according to the socioeconomic factors. Locker (2007) [7] observed that oral disorders had little impact on the health-related quality of life of higher income children but a marked impact on lower income children with ages of 11 to 14 years old. In this study we also found a highly significant association between low family income and negative impact on the OHRQoL of children with CP. De Camargo and Antunes [31] studied some socioeconomic factors in children with CP. They showed that there is a significant association between a low family income and high levels of untreated dental caries and need for dental treatment. This fact could lead to a poorer OHRQoL in these patients. It is, therefore, important to assess these conditions in general when dealing with oral health and OHRQoL in special care patients.

Although severity of communication ability, dental caries and low family income are associated with a negative impact on OHRQoL of children with CP, 43% of parents related that the overall well-being of the children is “not at all” affected by oral/orofacial conditions. This may occur because, in most cases, the children’s overall well-being is most affected by the severity of other clinical conditions of CP, rather than by the oral/orofacial condition itself.

A possible limitation of this study is that it was difficult to access all the CP clinics in the city. This resulted in a convenience sample of individuals who attended the dental treatment centre. Nevertheless whilst the lack of a control group makes it difficult to make generalizations about the findings of this study, it does raise awareness of potential differences in OHRQoL within the CP population. Another possible limitation of the study is the fact that parents answered the questionnaires (proxies) and it may not clearly reflect the children’s feeling and conditions.

It is important to assess the OHRQoL in all groups of patients, with the purpose to better understand the impact of diseases and socioeconomic factors in the patient’s lives, as well as to plan appropriate treatments according to the profile of each population.

## Conclusions

The severity of dental caries, severity of communication ability and low family income are conditions strongly associated with a negative impact on OHRQoL of children with CP. All these conditions should be assessed before medical and/or dental treatment of this population to prepare an appropriate treatment protocol and optimize oral health in these individuals.

## Competing interests

The author(s) declare that they have no competing interests.

## Author’s contribution

JA and TSC carried out the data collection, assisted by AOLO and ALC. MB and ALC initiated the idea and along with DR supervised the project and assisted in writing/editing of the article. All authors read and approved the final manuscript.

## Pre-publication history

The pre-publication history for this paper can be accessed here:

http://www.biomedcentral.com/1472-6831/12/15/prepub
